# Sex-specific differences in spring and autumn migration in a northern large herbivore

**DOI:** 10.1038/s41598-019-42639-3

**Published:** 2019-04-16

**Authors:** Lucie Debeffe, Inger Maren Rivrud, Erling L. Meisingset, Atle Mysterud

**Affiliations:** 1 Centre for Ecological and Evolutionary Synthesis, Department of Biosciences, University of Oslo, P.O. Box 1066 Blindern, NO-0316 Oslo, Norway; 2 CEFS, Université de Toulouse, INRA, 31326 Castanet-Tolosan, France; 3 Norwegian Institute of Bioeconomy Research, Department of Forestry and Forestry resources, NO-6630 Tingvoll, Norway

## Abstract

Ongoing global warming is now affecting migratory cycles in a large variety of taxa in seasonally variable environments. Disruption of migratory systems can cause population decline and affect ecosystem function across the globe. It is therefore urgent to understand the drivers of migration and how the different fitness limitations of the sexes affect migration, but studies seldom considered the full annual cycle. We analysed the annual migration cycle of 237 red deer (*Cervus elaphus*) in Norway and investigate how different seasonal limitations affected the variation in a suite of migration characteristics. We found fundamental differences in migration phenology between seasons, and migratory traits were much more variable in males. Spring migratory movements were characterized by longer distance roamed, lower speed, lasted longer, more frequent use of stopovers, timing was more synchronized and coincided with onset of plant growth, and with higher daily activity levels. Timing of autumn migration was more variable and not closely related to cease of plant growth. Our study emphasizes the benefits of studying the full annual cycle to gain further insight into the migration process, and how understanding the limitations of the full annual migration process of both sexes is critical for conservation purposes.

## Introduction

Migration between distinct seasonal ranges is widespread and commonly observed in taxa as different as invertebrates, fishes, birds and mammals^1^. Migration arises due to seasonal variation in limiting resources^2,3^ and allow individuals to track better foraging conditions or avoid predators, ultimately enhancing their fitness^1,2,4^. Ongoing global warming is known to affect migratory cycles^5,6^, and it is likely to affect ecosystem function across the globe^7^. A pattern common among long-distance migratory birds is that the timing of arrival in summer ranges is now earlier^8^, but nevertheless often mismatched relative to the peak availability of food in spring^9^. In large herbivores, there is currently focus on how well migrants track the “green wave” of high quality forage in early maturation stages in spring across the landscape^10,11^, and how this in turn depend on spring and early summer climate^12^. This tendency to focus on the importance of spring phenology is not unique to the migration literature. The climate change literature is currently biased towards studies performed during spring, largely neglecting the autumn season and therefore do not address how the characteristics of and drivers behind migration differ between the seasons^13,14^. This severely limits an understanding of the full annual migratory cycle^15^, as different processes affect different parts of the migratory cycle^16,17^. Current studies on the full migration cycle is biased towards birds with different feeding habit and social organization compared to large mammalian herbivores (e.g.^14,18^).

The focus on the spring season in the climate change and migration literature is to some extent understandable as spring is the time of reproduction, and assumed to have the strongest direct link to fitness. However, in chamois (*Rupicapra rupicapra*), it was recently demonstrated that onset of autumn shaped the timing of births more than onset of spring^19^, and mule deer (*Odocoileus hemionus*) overwinter survival was driven by both spring and autumn plant phenology^20^. Further, while forage maturation in spring plays a key role for timing of spring migration in ungulates^10,21^, it is clear that snow^22,23^ and onset of hunting season^24^ are important factors for timing of migration during autumn. In addition, the rutting season overlaps with the autumn migration season in many species, and onset of male migration in autumn is less linked to environmental stimuli compared to female autumn migration in red deer (*Cervus elaphus*)^24^. However, migration is rarely framed highlighting the different seasonal limitations on fitness of male compared to female ungulates^25^, and we lack an understanding of how different processes affect migration characteristics in spring versus autumn comparatively in male and female ungulates. In polygynous mammals, different factors limit male and female fitness^26,27^. This is framed in the reproductive strategy-predation risk hypothesis^28^ and the set of closely linked hypotheses connected to allometric scaling of body size relative to rumen size^29^, termed the body size^27^, nutritional needs^30^ or gastrocentric hypothesis^31^. The reproductive strategy-predation risk hypothesis states that males maximize their reproductive success by maximizing their body growth determining dominance rank and success in male-male combat during rut, while females should be risk-averse to maximize offspring survival^27^. Males are therefore expected to track the green wave closely during spring migration to optimize resources gain, while females are expected to be less risk prone seeking sheltered habitat. In contrast, from the body size and gastrocentric hypothesis, the larger males can survive on lower quality diet and not be expected to follow the green wave equally close. These hypotheses hence predict sexual differences in migration speed and duration, and in use of stopover sites (i.e. places where migratory individuals stop during the migration and before continuing their journey). However, most studies focus on the onset of migration^14,17,24,32–34^ or migration distances^35–38^. Other migration characteristics, like the duration of migration, speed or the use of stopover sites, are less studied for ungulates^11,39,40^, while it has been an important focus of studies on birds^15,41^. Since migratory individuals are exposed to an increasing number of threats during their journey^42^, a better understanding of the migratory movement for both spring and autumn migrations is critical for conservation^43^ and management purposes^44^.

In this study, we analyse an extensive dataset based on GPS locations of 237 male and female red deer in Norway. Earlier works on migration behaviour of red deer in Norway have shown that not all individuals are migrating, and that migratory individuals gain access to newly emergent plants and thus to higher quality diet by migrating between separate ranges compared to remaining in their winter ranges^11^. Plant phenology drives spring migratory behaviour in red deer, while onset of hunting is an important trigger of autumn migration^11,24^. In such context, we investigate how different seasonal and sex-specific limitations affected the variation in a suite of migration characteristics at the scale of the migration event (timing of departure, total distance roamed, duration of migration, movement speed, use of stopover sites) and using data on daily activity patterns. The current understanding of large herbivore migration in the northern hemisphere is that forage maturation can explain spring migration^10,11,21^, while autumn migration is about minimizing time in the more densely populated winter range, and simultaneously avoiding getting trapped by snow in the summer range^10,24^. We would therefore predict an overall slower spring migration with more stopovers and a more synchronised departure linked to onset of spring growth, while autumn migration is expected to be more directional and less well synchronised as it may be unrelated to forage conditions. With their larger body size, males can thrive on poorer quality forage than females^44^, and male migration behaviour is therefore predicted to be more variable and less tightly linked to forage maturation than female behaviour. Male migration in spring is then expected to be slower and lasting longer involving more roaming and a more extensive in use of stopover sites. Also due to their larger body size, males are less vulnerable than females to non-human predators due to their size^45,46^ and we predict that females, who are more vulnerable and less risk prone, to migrate quicker and more directed to limit time in unfamiliar terrain outside of seasonal home ranges. If rut determine male autumn movements, with males following females as a ‘resource’, we predict migration timing to be less variable for males than females, and to be quicker in autumn compared to spring, with a more direct route (i.e. a lower total distance roamed), a higher travel speed and a lower probability of using stopover sites (especially when departure is late).

## Results

To answer our question on seasonal and sex-specific variation in migration characteristics, we used linear or generalized linear mixed models and a model selection approach. Details on model selections and results from each best model can be found in the Supplementary Materials S3–S5. Only the selected models are presented below.

### Season and sex differences and variances of migration characteristics

**Figure 1:**
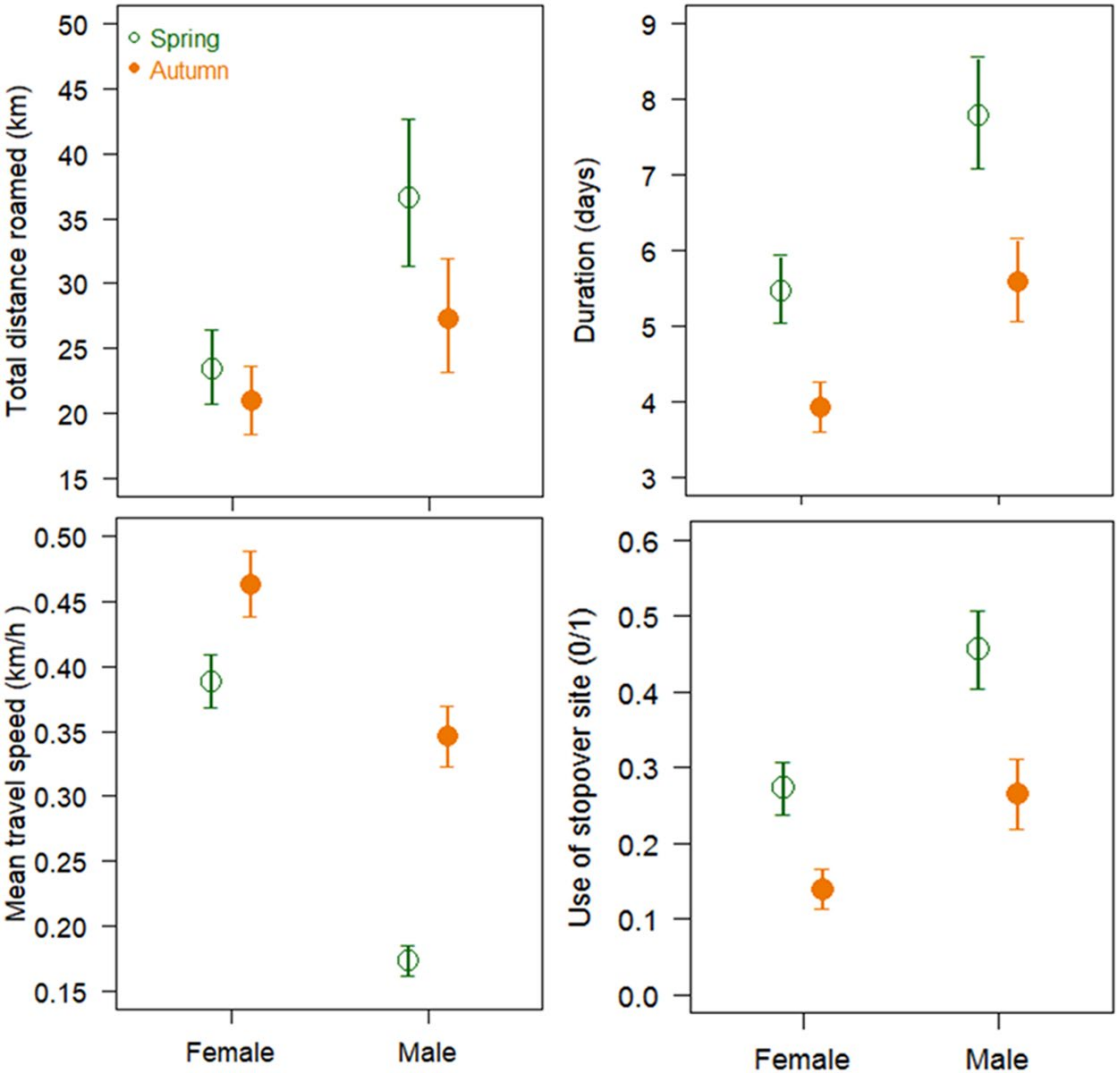
Estimated differences in migration characteristics (roaming distances, duration, mean travel speed and use of stopover site) according to the season and between sexes. The points depict relationships predicted by the selected models along with their corresponding standard error. The 4 migration movement characteristics were estimated using GPS locations from 237 migratory red deer across Western Norway from 2004 to 2015.

**Figure 2:**
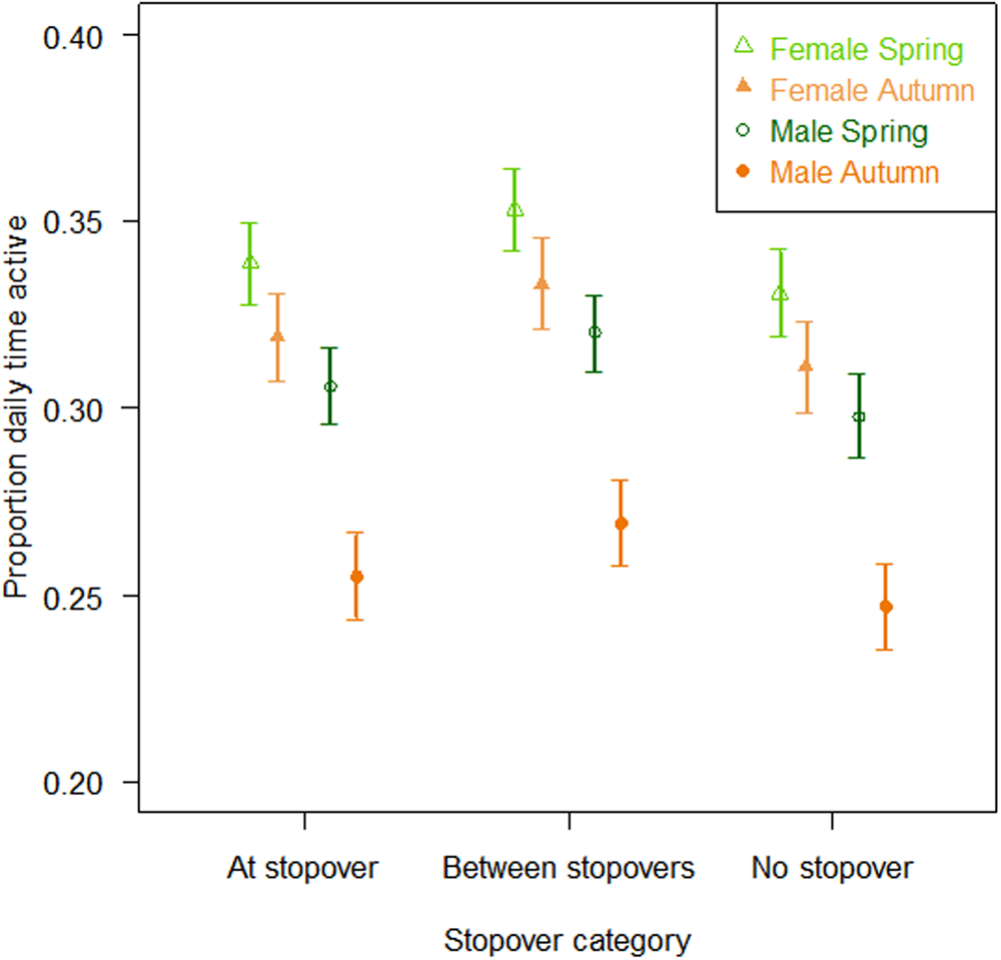
Estimated differences in proportion of time spent active during migration according to the season, sex and stopover use in the Møre & Romsdal county (which included 1 114 from the 2 447 daily mean activity counts). The points depict relationships predicted by the selected models along with their corresponding standard error. Activity patterns and stopover use were estimated using GPS locations and activity data from 237 migratory red deer across Western Norway from 2004 to 2015.

A summary of the data of the four migration characteristics according to sex and season are presented in Table 1. Season was included in all of the selected best models (sometimes as an interactive effect) investigating the migration characteristics distance roamed, use of stopovers and speed and duration of migration (Supplementary materials S3 and S4). Spring migratory movements were characterized by longer total distance roamed, lower speed, longer duration, more frequent use of stopovers (Table 2, Fig. 1), and higher daily activity levels (Table 2, Fig. 2). The variances of these migration characteristics in spring and autumn also differed significantly for three of the five characteristics in both females and in males. Timing of departure was more synchronised in spring in females only, while duration of migration and number of stopovers used were less variable during the autumn in both sexes (Table 3).

**Table 1:** Mean, minimum and maximum values of the 5 migration movement characteristics used as dependent variables in the analyses.

Number of recorded migration events	Female						Male					
	Spring			Autumn			Spring			Autumn		
	Mean ± SD	Min	Max	Mean ± SD	Min	Max	Mean ± SD	Min	Max	Mean ± SD	Min	Max
	N = 198			N = 159			N = 110			N = 82		
Timing of departure (Julian date)	124 ± 19	73	192	260 ± 33	164	354	123 ± 20	66	186	262 ± 25	192	348
Total distance roamed during migration (km)	35.80 ± 36.86	4.35	260.85	29.92 ± 27.72	3.83	201.34	64.07 ± 62.98	5.43	335.58	47.92 ± 51.94	3.30	295.86
Duration of migration (days)	8.17 ± 10.71	1	85	6.05 ± 10.03	1	93	15.08 ± 14.97	1	66	9.28 ± 14.15	1	100
Mean travel speed during migration (km/hour)	0.43 ± 0.20	0.06	1.40	0.51 ± 0.27	0.11	2.25	0.21 ± 0.16	0.05	1.20	0.46 ± 0.32	0.04	1.67
Number of stopover used during migration	0.41 ± 0.79	0	5	0.18 ± 0.40	0	2	0.77 ± 0.89	0	4	0.35 ± 0.62	0	3

Values are presented for both sexes and migration periods separately. The 5 migration movement characteristics were estimated using GPS locations from 237 migratory red deer across Western Norway from 2004 to 2015.

**Table 2:** Parameter estimates of the best models explaining variation in migration characteristics.

Model variable (fixed effect)	Departure timing			Distance roamed			Duration			Speed			Use of stopover			Daily activity levels		
	Estmt	SE	P	Estmt	SE	P	Estmt	SE	P	Estmt	SE	P	Estmt	SE	P	Estmt	SE	P
(Intercept)	−6.71	3.39	0.048	9.73	0.13	<0.001	0.05	0.19	0.78	−1.78	0.12	<0.001	−5.29	0.99	<0.001	0.36	0.01	<0.001
Sex _male_	—	—	—	0.28	0.12	0.025	0.35	0.08	<0.001	−0.31	0.07	<0.001	1.18	0.33	<0.001	−0.01	0.009	<0.001
Season _spring_	10.52	4.37	0.016	0.11	0.05	0.027	−0.05	0.20	0.796	0.26	0.12	0.032	−0.96	1.03	0.349	−0.06	0.01	<0.001
County _Møre & Romsdal_	17.37	4.17	<0.001	0.30	0.13	0.026	—	—	—	—	—	—	—	—	—	−0.02	0.01	0.183
County _Sogn & Fjordane_	4.67	4.96	0.346	0.20	0.15	0.196	—	—	—	—	—	—	—	—	—	0.01	0.02	0.357
County _Sør-Trøndelag_	6.67	5.06	0.188	0.62	0.17	<0.001	—	—	—	—	—	—	—	—	—	−0.02	0.01	0.12
Departure timing	—	—	—	—	—	—	0.01	0.004	0.003	−0.004	0.002	0.135	0.05	0.02	0.026	<0.001	<0.001	0.027
Distance between seasonal ranges	—	—	—	—	—	—	0.48	0.06	<0.001	0.37	0.04	<0.001	1.03	0.31	<0.001	—	—	—
Distance to coast	8.97	1.72	<0.001	—	—	—	0.06	0.04	0.122	—	—	—				—	—	—
Elevation difference during migration	—	—	—	0.13	0.03	<0.001	0.06	0.05	0.22	−0.01	0.03	0.805	0.64	0.16	<0.001	—	—	—
County _Møre & Romsdal_: season _spring_	−17.94	5.45	0.001	—	—	—	—	—	—	—	—	—	—	—	—	0.01	0.01	0.319
County _Sogn & Fjordane_: season _spring_	−6.98	6.60	0.29	—	—	—	—	—	—	—	—	—	—	—	—	−0.03	0.01	0.029
County _Sør-Trøndelag_: season _spring_	−11.55	6.72	0.087	—	—	—	—	—	—	—	—	—	—	—	—	0.03	0.01	0.019
Distance to coast: season _spring_	−7.53	2.28	0.001	—	—	—	—	—	—	—	—	—	—	—	—	—	—	—
Distance between ranges: season _spring_	—	—	—	—	—	—	0.14	0.07	0.047	−0.15	0.04	<0.001	0.76	0.35	0.029	—	—	—
Distance between ranges: Departure	—	—	—	—	—	—	−0.01	0.002	<0.001	0.003	0.001	0.002	−0.02	0.01	0.029	—	—	—
Departure timing: season _spring_	—	—	—	—	—	—	−0.01	0.003	0.006	—	—	—	—	—	—	<0.001	<0.001	0.001
Departure timing: sex _male_	—	—	—	—	—	—	—	—	—	0.005	0.002	<0.001	−0.03	0.01	0.006	—	—	—
Elevation difference: season _spring_	—	—	—	—	—	—	0.15	0.06	0.014	−0.11	0.04	0.002	—	—	—	—	—	—
Season _spring_: sex _male_	—	—	—	0.18	0.08	0.031	—	—	—	−0.51	0.07	<0.001	—	—	—	0.03	0.01	<0.001
Stopover use _Yes/In movement_	—	—	—	—	—	—	—	—	—	—	—	—	—	—	—	0.02	0.01	0.002
Stopover use _No_	—	—	—	—	—	—	—	—	—	—	—	—	—	—	—	0.01	0.01	0.266
Duration	—	—	—	—	—	—	—	—	—	—	—	—	—	—	—	0.01	0.01	0.231
Marginal and conditional R²	R²m = 0.08; R²c = 0.08			R²m = 0.13; R²c = 0.74			R²m = 0.37; R²c = 0.58			R²m = 0.46; R²c = 0.66			R²m = 0.38; R²c = 0.58			R²m = 0.14;R²c = 0.30		

Parameter estimates (Estmt), associated standard error (SE), and P-value of the selected generalized linear or linear mixed models explaining variation in migration characteristics. Deer identity and year were included as random factors in all models. The 5 migration movement characteristics were estimated using GPS locations from 237 migratory red deer across Western Norway from 2004 to 2015.

**Table 3:** Comparison of the variances between the two seasons (spring *versus* autumn) and sexes for each migration characteristics using a Levene’s test (*N*_female - spring_ = 233, *N*_male - sping_ = 118, *N*_female - autumn_ = 187, *N*_male - autumn_ = 89).

		Migration characteristic (*N* = *627*)	Female spring variance	Female autumn variance	Male spring variance	Male autumn variance	F	df	P-value	Fisher ratio of variance
Season variance differences	Female	Departure timing	334.46	1138.01	—	—	31.03	418	<0.001	0.29 [0.22 0.38]
		Distance roamed	0.65	0.55	—	—	2.65	418	0.104	1.17 [0.89 1.54]
		Duration	0.81	0.66	—	—	5.95	418	0.015	1.22 [0.93 1.61]
		Speed	0.24	0.23	—	—	0.004	418	0.947	1.04 [0.79 1.37]
		Number of stopover	0.57	0.16	—	—	13.88	418	<0.001	3.46 [2.63 4.46]
	Male	Departure timing	—	—	396.20	593.52	1.07	205	0.301	0.67 [0.45 0.98]
		Distance roamed	—	—	0.92	0.91	0.01	205	0.923	1.01 [0.68 1.49]
		Duration	—	—	1.15	0.82	6.42	205	0.010	1.39 [0.93 2.06]
		Speed	—	—	0.34	0.54	5.87	205	0.016	0.63 [0.43 0.94]
		Number of stopover	—	—	0.83	0.44	18.00	205	<0.001	1.88 [1.26 2.77]
Sex variance differences	Spring	Departure timing	334.46	—	396.20	—	0.78	349	0.377	0.84 [0.61 1.15]
		Distance roamed	0.65	—	0.92	—	4.81	349	0.029	0.71 [0.51 0.96]
		Duration	0.81	—	1.15	—	9.26	349	0.002	0.70 [0.51 0.96]
		Speed	0.24	—	0.34	—	4.32	349	0.038	0.71 [0.51 0.96]
		Number of stopover	0.57	—	0.83	—	18.31	349	<0.001	0.69 [0.50 0.93]
	Autumn	Departure timing	—	1138.01	—	593.52	5.46	274	0.020	1.92 [1.32 2.72]
		Distance roamed	—	0.55	—	0.91	9.34	274	0.002	0.61 [0.42 0.86]
		Duration	—	0.66	—	0.82	3.34	274	0.069	0.80 [0.55 1.13]
		Speed	—	0.23	—	0.54	22.43	274	<0.001	0.43 [0.30 0.61]
		Number of stopover	—	0.16	—	0.44	9.51	274	0.002	0.37 [0.26 0.53]

The 5 migration movement characteristics were estimated using GPS locations from 237 migratory red deer across Western Norway from 2004 to 2015.

A sex effect was included in most of the selected models investigating migration characteristics, highlighting differences in male and female migration (Supplementary materials S3 and S4). Females roamed shorter total distance than males (especially in autumn), but moved at higher speed (especially in spring, Table 2, Fig. 1). Their migration lasted fewer days and used fewer stopovers (except when later departure) (Table 2, Fig. 1), and had higher daily proportion of time spent active (Table 2, Fig. 2). The variances of migration characteristics in males and females differed significantly for four of the five characteristics in both seasons (Table 3). Females showed lower variances in their migration characteristics than males, except for timing of departure in spring and duration of migration in autumn. Timing of departure in autumn was more synchronised for males (Table 3).

### Landscape and movement covariate effects on individual migration characteristics

Each selected model investigating the effect of landscape and movement on the migration characteristics (i.e., distance roamed, timing of departure, speed and duration of migration, use of stopovers and time spent active) included different sets of landscape variables (Table 2). Timing of departure, total distance roamed and daily proportion of time spent active differed according to the county of capture and the magnitude of the effect depended on the season. Individuals having their winter home range 28.0 km further away from the coastline left their seasonal range 1.4 and 10.0 days later in spring and autumn, respectively, and spent 0.4 more days (corresponding to a 4.4% and 10.0% increase of the mean and median migration duration, respectively) in migration (Table 2; Supplementary material S5). A 550.0 m increase in absolute difference in elevation between seasonal ranges increased the total distance roamed during migration of 7.8 km, led to 3 and 0.5 days longer spring and autumn migrations, respectively. It further decreased migration travel speed by 0.1 km/h in spring, by 0.01 km/h in autumn and increased the probability of using stopovers by 15.0% in spring (Table 2; Supplementary material S5). Note that the marginal r² for the selected models for departure timing and total distance roamed during migration were low (r^2^ = 0.08 and 0.13 respectively; Table 2).

The use of stopover sites decreased with later departure in males only, indeed a 87.0% decline of the use of stopover site was found throughout the migration period, while the females use of stopover remained stable (Table 2; Supplementary material S5). Moreover, individuals using stopovers showed 2.2% higher activity when traveling compared to individuals not using stopover sites (Table 2; Fig. 1). In spring, the migration duration decreased by 2.0 days and the proportion of daily time spent active increased by 0.4% as the timing of departure became 10.0 days later, while it decreased only by 0.8 day and 0.3% in autumn migration (Table 2; Supplementary material S5). Increasing distance between seasonal ranges led to a higher probability to use the stopover site, to faster migration speed in autumn and for individuals with later departure, but to longer migration duration in spring and for individual leaving early (i.e. a linear distance between seasonal ranges increasing of 10.0 km lead to a 5.2 days longer migration and increased the probability of using stopover sites by 17.4% in the spring; Table 2).

## Discussion

Despite the huge amount of work on migration, there are few extensive analyses of how the migration phenology differs between sexes during spring and autumn. Despite the extensive theory for sexual differences in ecology of polygynous mammals^27^, we know little about how the different fitness limitations experienced by the sexes affect migration. We found marked differences in migration patterns both between seasons and between sexes as predicted from these bodies of theory, emphasizing the need for comprehensive studies expanding the horizon beyond season- and species-specific focus, and instead including the full migratory cycle on the level of the sexes. This is necessary to make informed predictions of impacts of future climate change on migratory species, and for developing suitable mitigation and conservation efforts. Indeed, since the drivers of migration are likely to differ between males and females, and lead to varying migration behaviour between sexes among seasons, sexes may differ in their ability to cope with climate change and adjust their migratory behaviour to climate and environmental conditions^47,48^. In such context, implementing management or conservation plans that account for seasonal sex differences would be crucial for success. Although sex differences in bird migration is widely acknowledged as important^14,18,34,47,49^, the patterns are expected to be different for mammals compared to birds due to different mating seasons and mating systems. While migratory birds arrive and mate in spring, ungulates mate in autumn and give birth in spring with only females taking care of the young. Our results highlight some fundamental differences in the migration phenology and characteristics between spring and autumn migratory movements in red deer, as season was included in all the selected models. Spring migration was more constrained by plant phenology than autumn migration, the timing of departure in spring was less variable in females, but duration and use of stopovers were more variable during spring compared to autumn. There were also marked sex differences in migration characteristics in the same landscapes, highlighting how the different factors limiting fitness in males and females also affect migration. Moreover, both season and sex were important factors modulating the effect of environmental and movement covariates (distance between seasonal ranges, elevation difference during migration, and winter range distance to coast).

### Autumn and spring migration phenology and characteristics

**Figure 3:**
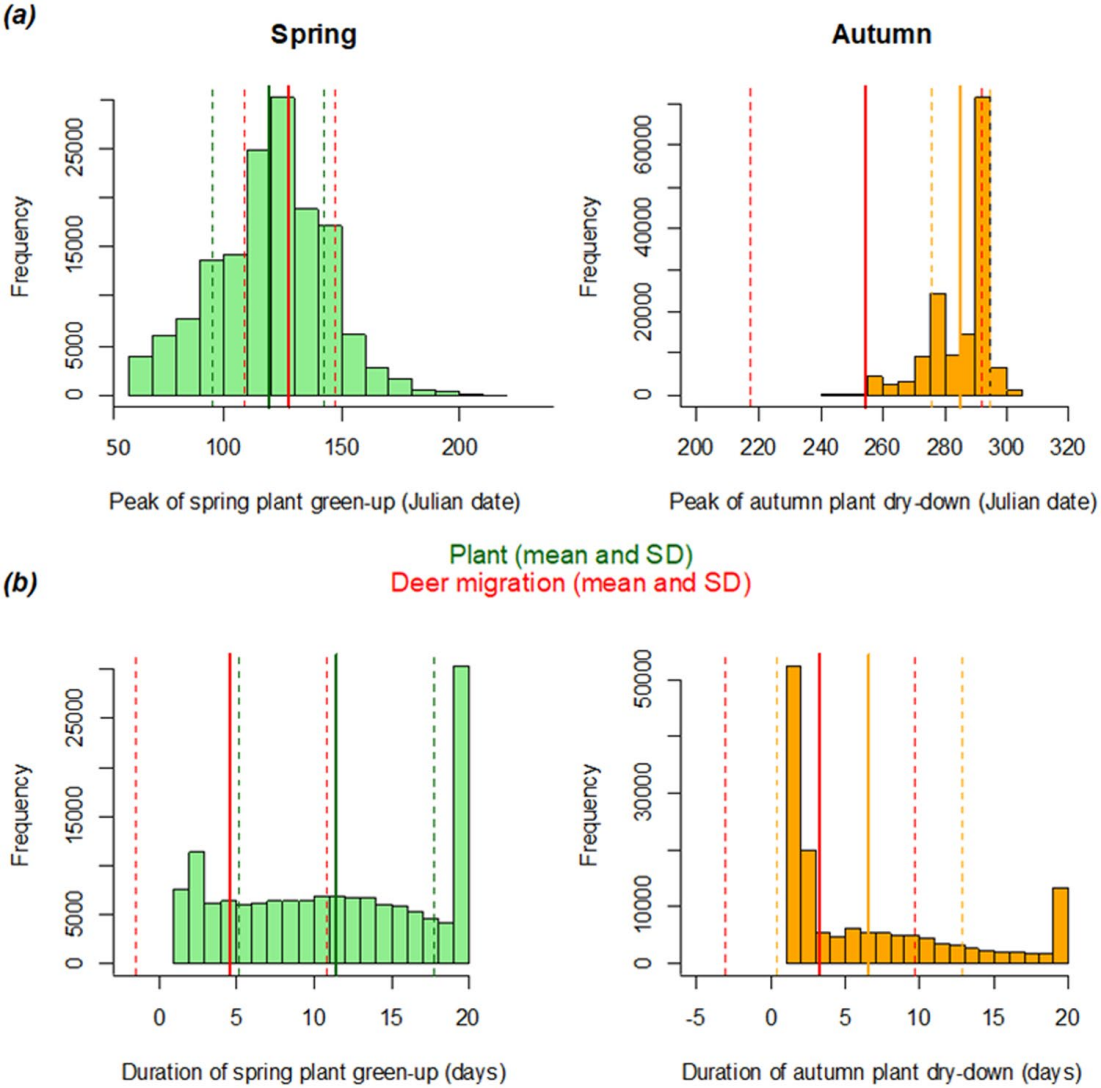
Distribution of the plant phenology parameters (**a**) peak and (**b**) duration in spring (green) and autumn (orange) for each pixel of the study area visited by a deer (*N* = 147 438) according to deer migration timing. The green and orange lines represent (**a**) the mean Julian date of peak spring green-up and autumn dry-down, respectively and (**b**) the mean duration of spring green-up and autumn dry-down, respectively, over the course of the study period. Red lines represent (**a**) the mean mid-date of migration and (**b**) the mean duration of migration. Dashed lines represent the standard error around the mean. Deer migration timing was estimated using GPS locations from 237 migratory red deer across Western Norway from 2004 to 2015.

Autumn and spring migration drivers can differ substantially^15,24^, potentially leading to different phenology and characteristics^50^. For instance, white-fronted geese (*Anser albifrons*) had a more in-depth use of stopover sites to acquire extra energy stores during spring than autumn^15^. There is solid evidence that ungulates are surfing the green wave during spring migration^10–12,51–53^, but no such link with plant phenology was found in the autumn^24,53^ (Figs 2, 3). Indeed, in the same population, it has been found that onset of hunting season was an important trigger of autumn migration, while snow fall played a minor role^24^. We found that migration was more synchronous and slower in spring while, at a broad scale, plant phenology was more synchronous and shorter in autumn. This discrepancy highlights the lack of a link between timing of red deer migration and plant phenology in autumn. Earlier studies have found that autumn migration seem to be a trade-off decision between reaching the winter ranges before being trapped by snow on one side, and avoiding spending longer time in the winter range with higher population densities^16,32,53^. These different processes are the likely drivers behind the different migration patterns in the two seasons in ungulates. We found that the duration of migration was longer for early departure individuals, especially in the spring, but surprisingly, individuals roaming longer distances did not leave their seasonal ranges earlier (Supplementary material S2). Individuals roamed longer total distance during their spring migration (suggesting a less straight trajectory) and moved at a lower speed. These characteristics, as well as the higher daily activity levels found in spring, are pointing towards a more thorough use of the migration corridor during spring compared to autumn. In some areas, the time spent on spring migration are critical for obtaining resources^15,39^, though red deer in Norway spend a rather short amount of time en-route^11^. A more synchronized departure timing in spring compared to autumn was also found in several roe deer (*Capreolus capreolus*) populations^22^, wildebeest (*Connochaetes taurinus*)^54^, and African bush elephant (*Loxodonta africana*)^55^. On the other hand, hunting may influence autumn migration^24^ so the individuals limit their exposure to hunting (e.g. shorter duration, higher travel speed, shorter total distance roamed and fewer stops at stopover). As the phenology of plants both in spring and autumn is predicted to change under future climate warming, our results show that migration can be impacted differently depending on both season and sex. Therefore, year round climate change vulnerability assessments are required to inform management and conservation decisions regarding migrant species^6^.

### The use of stopover sites

The ecological importance of stopover sites during migration has been highlighted in numerous studies in birds^15,41^, but more seldom in ungulates^39^. In mule deer (*Odocoileus hemionus*), the use of stopover sites during migration was more widespread during the spring migration than in autumn^39^. They also found that the forage quality of the stopover sites increased with elevation and distance from winter range. A similar link with forage quality in our system may explain the positive relationship between the probability of using stopover sites in spring and elevation gain during migration, and with distance between the seasonal ranges. On a daily scale, individuals using the stopover sites showed higher activity levels when travelling compared to individuals not using stopovers. At the stopover sites however, there was no difference in activity. This suggests the use of two contrasting strategies by the migratory deer; moving slower using the migratory corridor to feed on the way without using the stopover sites, or moving faster in the migratory corridor thus jumping between stopover sites. These results provide further evidence for the importance of conserving stopover sites along the migration corridors^39^.

### The role of landscape

Several aspects of migration are known to be affected by landscape features^38,56,57^. For instance, migration distance in terrestrial mammals has been linked to resource availability, with animals living in resource-poor environments travelling farther to fulfil their resource needs^36^. In our study, elevation differences experienced during migration was an important landscape feature. Indeed, both the duration of migration and the probability of using stopover sites increased as elevation gain increased during migration (with a steeper slope during spring migration for duration), and the speed of migration decreased with increasing elevation gain in spring; suggesting that increased energy expenditure of locomotion or following the snow melt closer might play a role. The higher probability of using the stopover sites might then depend on the higher availability of plants at different growth stages the individual experienced, as elevation gain increased with a more variable snow melt^53^. Winter range location is also of importance when explaining the variation observed in the different migration characteristics, since the county of capture and/or the distance of its winter home range barycenter to coastline were included in some of the selected models. For instance, individuals having winter ranges further from the coastline spent more time migrating and left their summer range later. Elevational range shifts due to warming was observed in some mountain ungulates, but not all, in the Swiss Alps^58^. Based on our results, it is likely that it may also differ between sexes in some species. This highlights the importance of better documenting the ability of ungulates in different landscapes to cope and mitigate future impact of climate change.

### Sex differences in migration phenology and characteristics

In polygynous mammals, the sexes have very different constraints on fitness^26^, leading to largely different ecology of males and females^27^. Different activity budgets between the sexes are widespread and also an important mechanism to explain social segregation^59,60^. Even if males and females respond similarly to the onset of phenological changes in plant development in the spring^11^, at a finer scale their migration characteristics and daily activity patterns differ. Indeed, timing of departure was similar between sexes, contrary to what was predicted under the body size or gastrocentric hypothesis predicting that males’ migration is less tightly connected to plant phenology^44^. At a finer scale, male migration lasted longer, with increasing total distance roamed during migration^38^. Males did show a higher variability in migratory characteristics, giving some support to both the gastrocentric hypothesis, and their lower vulnerability to non-human predators^31,46^. In contrast, females migrated at higher speed, especially during spring and when leaving their seasonal range early, possibly to reach their summer range before upcoming parturition. At the daily scale, females were more active than males during both spring and autumn migrations, which is consistent with the higher female activity previously reported during the main growth season^25^. The use of stopover sites also differed between sexes, with a decreasing probability of using stopovers with later departure timing in males, while a weaker effect was found in female with the reverse trend, also suggesting different constraints and responses. Males and females also differed in terms of variability of the migration characteristics. In our study, even if timing of migration did not differ between sexes in red deer, the synchronicity of departure differed, together with the remaining migration characteristics investigated. In contrast to birds that both are mating and arriving in spring, ungulates with rutting season in autumn had markedly different phenology in males and females. Indeed, intra-individual variation was lower in females during both spring and autumn for all the migration characteristics explored, except for timing of departure; suggesting that females are more constrained in their migratory behaviour, likely because of gestation or calving in spring and the presence of offspring during autumn^26^. Males were far more synchronized than females in autumn migration departure timing, highlighting the possible effect of the start of the rutting season on male migratory behaviour. The quicker male migration found in autumn, with a higher travel speed, a more direct route (i.e. shorter total distance roamed), and a low probability to use stopover sites when departing late, also indicate a possible effect of the start of the rutting season on male migratory behaviour in autumn, with males tracking females as a ‘resource’. However, no sex differences were found for variability in spring migration departure, contrary to what was found in moose and roe deer^22,38^. In birds early arrival in spring and longer residency in autumn likely yield benefits for males defending their breeding territory^14,34^, but sex-specific patterns are variable between species^14,18,34^.

Our study on the full annual cycle of migration in both males and females, using 6 migration characteristics, highlight how the different seasonal limitations interact with the differing life histories of the sexes. Our results show that spring migration is more constrained by changes in plant phenology than autumn migration, and the higher variability in migratory traits found in males than females could imply a higher tolerance for males to the predicted increase in climate variability^61^. Given the increasing number of threats migratory species are exposed to^6,42^, and the observation that disruption of migratory routes can causes population collapse^3^, a better understanding of the full migration process is critical to predict future responses of ungulates to global change.

## Methods

### Study area

The study area covered the main distribution range of red deer on the west coast of southern Norway (counties of Hordaland, Sogn & Fjordane, Møre & Romsdal and Sør-Trøndelag). The area ranges across different landscapes and topography, from flatter coastal areas to high mountains and valleys inland, with the fjord landscape in between (Supplementary material S1). Forest vegetation is dominated by deciduous species, Scots pine (*Pinus sylvestris*) and planted Norway spruce (*Picea abies*). For a more detailed description of the study area, see^16^. The study area was divided in several management units. These management units mainly reflect landowner boundaries and history rather than biological populations^44^.

### Red deer movement and migration characteristics

Between 2004 and 2015, adult red deer (females ≥ 1.5 years; males ≥ 2.5 years) were captured mainly during winter (January to May), individually tagged and fitted with GPS-collars (Televilt/Followit, Stockholm, Sweden and Vectronic, Berlin, Germany) scheduled to take a GPS position every second hour. Data from the first 24 hours after marking were discarded and the raw data was screened for outliers following Bjørneraas *et al*.^62^. Individual space use tactic (migratory, 55.1% or resident, 44.9%) was determined using the Net-Square Displacement (NSD) technique developed by Bunnefeld *et al*.^40^, and adapted by Bischof *et al*.^11^ so that individual fit was assessed manually. This method is well developed within our study system, and has proven to work well on assessing migration behaviour in our previous work^11,16,24^.

As detailed in our previous work^11,16^, we fitted separate logistic curves to each migratory movement of the 247 migratory deer to estimate the distance between seasonal ranges (i.e. asymptote parameter), the mid-date of migration (i.e. inflection point) and the time needed from mid-migration date until 75% of the asymptote has been reached (i.e. scale parameter). Based on these estimates, we then calculated the timing of departure (defined as mid-migration date −2 × scale parameter), the duration of migration (defined as [mid-migration date +2 × scale parameter] −[mid-migration date −2 × scale parameter]), the total distance roamed (defined as the sum of the Euclidian distances between successive locations during migration), the mean speed of travel (defined as the mean speed between two successive locations during migration) and the number of stopover sites used during migration for both spring (*N* = 351) and autumn (*N* = 276) migration. Stopover sites were identified as the highest 25% quantile in the utilization distribution along each migratory trajectory estimated using Brownian bridge movement models^11,43^, and the identified stopovers along the migration route were assessed visually.

All applicable institutional and/or national guidelines for the care and use of animals were followed and we confirm that all experiments were performed in accordance with relevant guidelines and regulations. All red deer capture and marking procedures have been approved by the Norwegian Animal Research Authority, chemical immobilization and marking follow standard protocols^63^.

### Daily activity data

Sixty-two percent of the collars included a dual-axis acceleration sensor that counts both horizontal and vertical individual neck movements, allowing to discriminate active (i.e., all behaviour confounded) and inactive time. The proportion of daily time active was calculated as the number of activity values set as active (ie., above a threshold value) during a day divided by the total number of values obtained on that day^25^. Days with less than five activity values were discarded. Daily data on activity was available for 148 migratory deer.

### Environmental characteristics

Digital maps providing distance to coastline (in kilometres), and elevation (m a.s.l.; provided by the Norwegian Mapping Authority) were used to extract these characteristics at the home range scale (i.e., for the summer and winter ranges, and excluding the migratory trajectory). All maps were rasterized with a resolution of 100 × 100 m^11^. The difference in elevation resulting from the migratory movement was calculated as the absolute difference in elevation between the last location at the winter or summer range and the first location at the summer or winter range, for spring and autumn migrations respectively (seasonal ranges were estimated using the 95% fixed kernel density estimator with an ad hoc method used for the smoothing parameter). In order to compare the duration and synchrony of plant phenology between spring and autumn at the large scale, we used the satellite-derived vegetation index NDVI (normalized difference vegetation index^64^) as a proxy of forage quality and quantity to quantify the rate of green-up in spring and dry-down/forage deterioration in autumn for each pixel visited by a migrant deer^11,24^. NDVI measures overall greenness, and does not discriminate between understory growth and forest vegetation. However, as a good link between ungulate forage quality and NDVI have been established^21,65,66^, and strong signals have been found when relating NDVI to red deer migration on a similar scale in earlier studies^11^, NDVI is considered a reliable proxy of ungulate forage quality in this study. NDVI images from the MODIS TERRA satellite were provided by NASA (http://modis.gsfc.nasa.gov/) with a temporal resolution of 16 days and a spatial resolution of 250 × 250 m. The raw NDVI time series were processed, subsampled and modelled according to Bischof *et al*.^11^ and adapted by Rivrud *et al*.^52^, where the end product is an annual double logistic curve of NDVI values for each pixel visited by the red deer. The instantaneous rate of green-up (IRG) in spring and the instantaneous rate of dry-down (IRD) in autumn is calculated by taking the first derivative of the spring- and fall-part respectively of the double logistic NDVI curve^11,52^. The IRG and IRD values are then connected to the red deer GPS data. Duration and synchrony of plant phenology changes differed between spring and autumn. The vegetation green-up took 11.45 days (SD = ±6.27) from onset to peak in spring, while the vegetation dry-down took 6.57 days (±6.19) in autumn (Student’s t-tests: *N* = 156 424 pixel, *t* = 212.37, df = 294 790, *P* < 0.001; Fig. 3). Further, vegetation green-up was less synchronous than vegetation dry-down (standard deviation of the peak of green-up or dry-down Julian date, respectively: 23.64 and 9.63 days; Levene’s test: *N* = 156 424 pixel, df = 294 874, *F* = 68 437, *P* < 0.001; Fisher ratio of variance = 6.03 [5.97 6.09]; Fig. 3).

### Data analysis

Some individuals were removed due to missing values in the covariates, 237 individuals were then available for analyses on migration characteristics (including 309 spring migration events and 241 autumn migrations events) and 147 on daily activity pattern (including 185 spring migration events and 147 autumn migrations events).

#### Variance analyses

To test if the variances of the 5 migration characteristics (timing of departure, total distance roamed during migration, duration, mean travel speed and number of stopovers used during migration) differ between seasons (spring *vs*. autumn) and sexes we used Levene’s tests implemented in the R package ‘car’^67^. Levene’s tests allow us to test our predictions that migration timing will be less variable for males than females if linked to our hypothesis that rut determines male autumn movements, and that migration departure will be more synchronised if linked to onset of spring growth, while autumn migration is expected to be less well synchronised as it may be unrelated to forage conditions. Variables were log-transformed to optimize normality (see Supplementary material S2 for distributions), but since the normality conditions were not always fully met, the more robust Levene’s test was used instead of the more commonly used Fisher test.

#### Model structure – migration characteristics

To answer our question on seasonal and sex-specific variation in migration characteristics, we used linear or generalized linear mixed models implemented in the R package ‘lme4’^68^. Specifically, we modelled the timing of departure (defined as the individual Julian date of departure–the median Julian date of departure of the corresponding season), the total distance roamed during migration, the duration, mean travel speed and the use of stopovers (binary variable) according to the season (spring *vs*. autumn), sex, county, distance of the winter home range barycenter to coastline (defined as the Euclidean distance between the coastline and the average location from all the winter GPS locations), absolute difference in elevation during migration, distance between seasonal ranges (except for models on total distance roamed) and timing of departure (except for models on timing of departure). Because patterns were expected to be sex- and season- specific, two-way interactions with sex and season were considered for all variables. For models on duration, speed and use of stopovers the two-way interaction between distance between seasonal ranges and timing of departure was also included.

#### Model structure – daily features

To investigate potential differences during migration on a daily scale, we modelled the proportion of daily time active according to the use of stopovers (3 categories: no use of stopover, use of stopover - days at stopover, use of stopover - days between stopovers), season (spring *vs*. autumn), sex, county, distance of the winter home range barycenter to coastline, total distance roamed during migration, timing of departure and duration. Patterns were also expected to be sex- and season- specific, so two-way interactions with sex and season were considered for all variables (except for the interactions between duration and season and total distance roamed and season to avoid over-parametrization of the full model).

Individual identity and year were included as random intercepts in all models to account for unbalanced sample size. Since the type of activity data differed depending on collar brand this factor was included in the models explaining the proportion of daily time active. All variables were rescaled and/or transformed when necessary to optimize estimation^69^. The total distance roamed during migration, the duration and mean speed were log-transformed, the absolute difference in elevation during migration, the distance between seasonal ranges and departure timing were rescaled by centring on the mean and dividing by the standard deviation, and the proportion of daily time active was transformed using an arcsin (x*2/π) function allowing the results to be rescaled between 0 and 1 (see Supplementary material S2 for distributions).

#### Model selection

To avoid correlation issues we checked that all variables included in the same model were not correlated more than a conservative threshold of *r* = 0.3^69^. As the total distance roamed and duration of migration were highly correlated (Pearson’s product-moment correlation: correlation = 0.83, *n* = 5665, *t* = 113.26, *df* = 5663, *P* < 0.001), these factors were not included in the same model. We fitted the global models described above as well as all simpler derived models in R using the AICcmodavg package^70^. The best models were then selected using the Akaike Information Criterion corrected for small sample size (AIC_*c*_), which reflects the best compromise between model precision and accuracy^71^. According to the rule of parsimony, we selected the simplest model within 2 AIC*c* of the top model.

## Supplementary information

Supplementary materials

## Data Availability

All data supporting the results will be made available on Dryad digital repository in due time and are available from the corresponding author on reasonable request at anytime.
