# Supplementary material for: Sex-specific differences in spring and autumn migration in a northern large herbivore
Source: Sci Rep. 2019 Apr 16;9:6137. doi: 10.1038/s41598-019-42639-3 (PMC6468013; doi:10.1038/s41598-019-42639-3)
Supplement: Supplementary file 1 — Supplementary materials [file 41598_2019_42639_MOESM1_ESM.docx]

**Sex-specific differences in spring and autumn migration in a northern large herbivore**

**Lucie Debeffe^1,2*^, Inger Maren Rivrud^1^, Erling L. Meisingset^3^, Atle Mysterud^1^**

^1^ Centre for Ecological and Evolutionary Synthesis, Department of Biosciences, University of Oslo, P.O. Box 1066 Blindern, NO-0316 Oslo, Norway

^2^ CEFS, Université de Toulouse, INRA, 31326 Castanet-Tolosan, France

^3^ Norwegian Institute of Bioeconomy Research, Department of Forestry and Forestry resources, NO-6630 Tingvoll, Norway

**Supplementary materials**

***S1. Map of the study area***

***S2. Response variable distributions and correlations***

***S3. Model selection procedures***

***S4. Predictor weights***

***S5. Result section – details***

***S6. Use of a Multivariate ANalyses Of Variance (MANOVA)***

**Appendix - S1. Map of the study area**

***
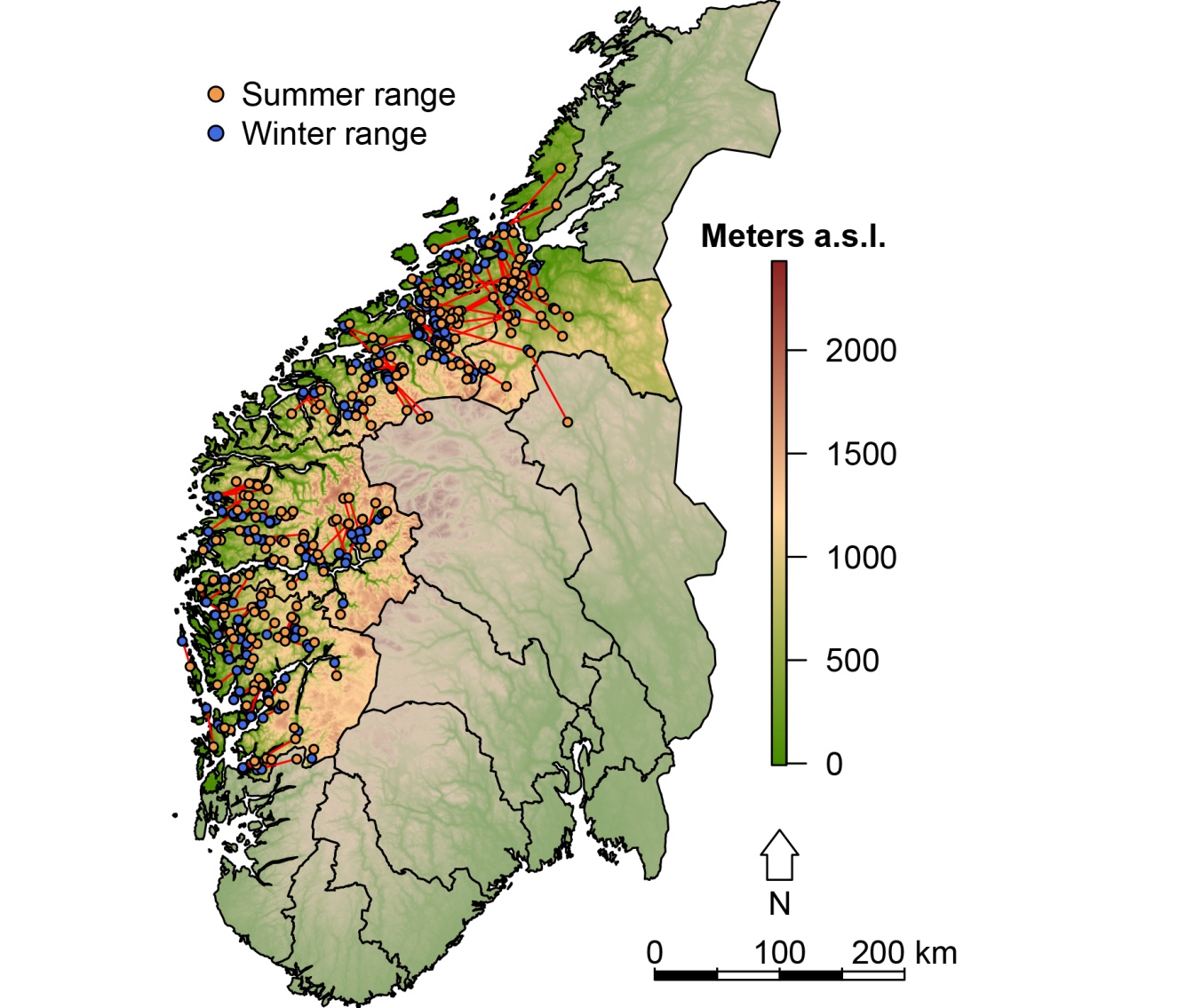
***

**Fig S1.1.** Map of southern and central Norway showing the gradient of elevation and each deer’s c summer and winter range center (in orange and blue, respectively). Each individual’s home ranges are linked by a red line. The four counties of the study area are shown in brighter colors. The map was drawn using R software (R Core Team (2018). R: A language and environment for statistical computing. R Foundation for Statistical Computing, Vienna, Austria. URL https://www.R-project.org/.)

***Møre & Romsdal***

***Sogn & Fjordane***

***Sør-Trøndelag***

***Hordaland***

**Appendix - S2. Response variable distributions and correlations**


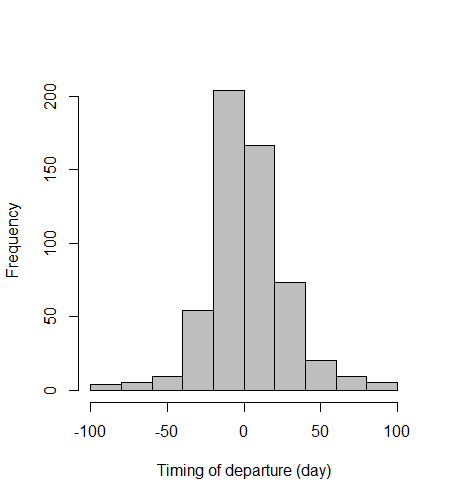
**
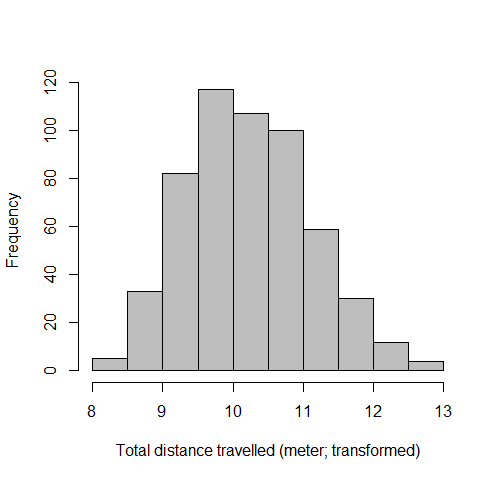
**
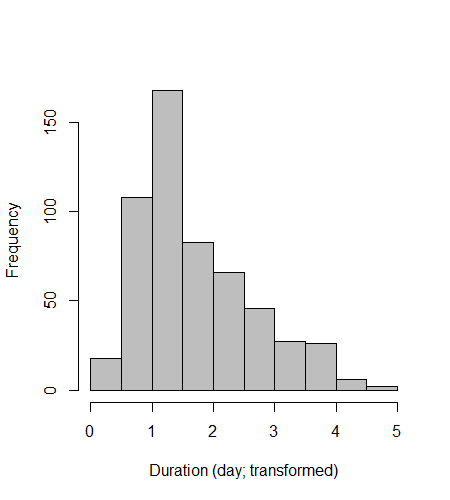
**
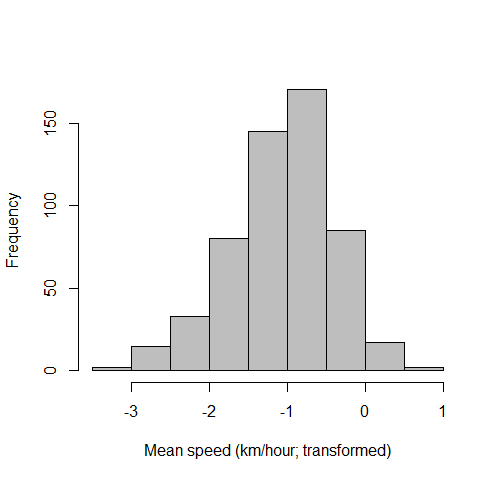
**
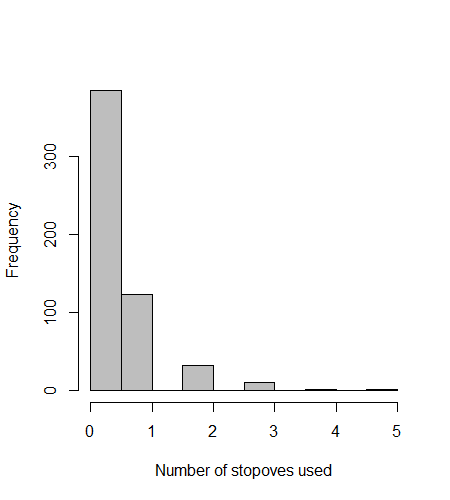


(d)

(c)

(e)

(b)

(a)

**Fig S2.1.** Distribution of the 5 migration movement characteristics (*N* = 550): (a) timing of departure, (b) total distance roamed during migration (log-transformed), (c) duration of migration (log-transformed), (d) mean travel speed during migration (log-transformed) and (e) number of stopover used during migration (log-transformed).


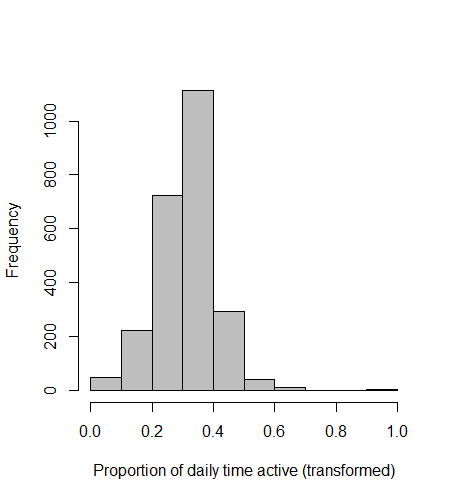


**Fig S2.2.** Distribution of the transformed response variables used to study the daily pattern of migration; the proportion of daily time active (*N* = 2447; transformed using an arcsin (x*2/π) function).

**Table S2.1.** Correlation value between migration characteristics with associated P-value.

| **All migration events (N=627)** | Euclidean distance between seasonal ranges | Number of stopover sites used | Duration | Total distance roamed | Timing of departure |
| --- | --- | --- | --- | --- | --- |
| Number of stopover sites used | 0.36 (P < 0.001) | - |  |  |  |
| Duration | 0.49 (P < 0.001) | 0.73 (P < 0.001) | - |  |  |
| Total distance roamed | 0.77 (P < 0.001) | 0.62 (P < 0.001) | 0.80 (P < 0.001) | - |  |
| Timing of departure | -0.06 (P = 0.15) | -0.10 (P = 0.012) | -0.14 (P < 0.001) | -0.06 (P = 0.15) | - |
| Mean travel speed | 0.20 (P < 0.001) | -0.35 (P < 0.001) | -0.48 (P < 0.001) | 0.004 (P = 0.92) | 0.14 (P < 0.001) |

**Appendix - S3. Model selection procedures**

**Table S3.1.** Model selection tables for the five migration characteristics (*N* = 550): (a) timing of departure, (b) total distance roamed during migration, (c) duration of migration, (d) mean travel speed during migration and (e) use of stopover. The full model included fixed effects of the season (spring *vs.* autumn), sex, county, distance of the winter home range barycenter to coastline (‘Dcoast’), absolute difference in elevation between seasonal ranges (‘ElevDiff’), distance between seasonal ranges (‘DS&W’; except for models on total distance roamed) and timing of departure (‘Timing’, except for models on timing of departure), and their two-way interactions with sex and season. For models on duration, speed and use of stopover the two-way interaction between distance between seasonal ranges and timing of departure was also included. In addition individual identities and years were added as random factors in all models. The loglikelihood (LogLik), corresponding AIC*c* weight (AIC*cW*) are presented for all models with a ΔAIC*c* < 2.5. The candidate model sets (i.e., models with a ΔAIC*c* < 2) are in grey shade and the selected models are shown in bold.

| **(a) Models explaining timing of departure** | **df** | **logLik** | **AICc** | **∆AICc** | **AICcW** | **CumW** |
| --- | --- | --- | --- | --- | --- | --- |
| **County : Season + Dcoast : Season** | **13** | **-2526.93** | **5080.50** | **0.00** | **0.21** | **0.21** |
| DS&W + County : Season + Dcoast : Season | 14 | -2526.11 | 5081.00 | 0.46 | 0.17 | 0.37 |
| ElevDiff : Season + County : Season + Dcoast : Season | 15 | -2525.25 | 5081.40 | 0.86 | 0.14 | 0.51 |
| DS&W + ElevDiff : Season + County : Season + Dcoast : Season | 16 | -2524.51 | 5082.00 | 1.51 | 0.10 | 0.60 |
| ElevDiff + County : Season + Dcoast : Season | 14 | -2526.75 | 5082.30 | 1.75 | 0.09 | 0.69 |
| Sex + County : Season + Dcoast : Season | 14 | -2526.91 | 5082.60 | 2.06 | 0.07 | 0.77 |
| DS&W + County : Season + DS&W : Season + Dcoast : Season | 15 | -2525.97 | 5082.80 | 2.31 | 0.07 | 0.83 |
| ElevDiff + DS&W + County : Season + Dcoast : Season | 15 | -2526.04 | 5083.00 | 2.44 | 0.06 | 0.89 |
| DS&W + Sex + County : Season + Dcoast : Season | 15 | -2526.06 | 5083.00 | 2.49 | 0.06 | 0.95 |

| **(b) Models explaining the total distance roamed during migration** | **df** | **logLik** | **AICc** | **∆AICc** | **AICcW** | **CumW** |
| --- | --- | --- | --- | --- | --- | --- |
| County + ElevDiff : Season + Season : Sex | 12 | -545.07 | 1114.70 | 0.00 | 0.12 | 0.12 |
| County + ElevDiff : Season + Season : Sex + Season : Timing | 14 | -543.22 | 1115.20 | 0.50 | 0.09 | 0.21 |
| County + Dcoast + ElevDiff : Season + Season : Sex | 13 | -544.46 | 1115.60 | 0.88 | 0.08 | 0.29 |
| County + Dcoast + ElevDiff : Season + Season : Sex + Season : Timing | 15 | -542.56 | 1116.00 | 1.31 | 0.06 | 0.35 |
| County + ElevDiff : Season + Season : Sex + Season : Timing + Dcoast : Season | 16 | -541.67 | 1116.40 | 1.65 | 0.05 | 0.40 |
| **ElevDiff + County + Season : Sex** | **11** | **-546.95** | **1116.40** | **1.68** | **0.05** | **0.45** |
| County + ElevDiff : Season + Season : Sex + Dcoast : Sex | 14 | -543.96 | 1116.70 | 2.00 | 0.04 | 0.49 |
| County + Timing + ElevDiff : Season + Season : Sex | 13 | -545.06 | 1116.80 | 2.08 | 0.04 | 0.54 |
| County + ElevDiff : Season + ElevDiff : Sex + Season : Sex | 13 | -545.07 | 1116.80 | 2.10 | 0.04 | 0.58 |
| County + ElevDiff : Season + Season : Sex + Dcoast : Season | 14 | -544.02 | 1116.80 | 2.10 | 0.04 | 0.62 |
| County + ElevDiff : Season + Season : Sex + Season : Timing + Timing : Sex | 15 | -542.97 | 1116.80 | 2.13 | 0.04 | 0.66 |
| County + Sex + ElevDiff : Season | 11 | -547.25 | 1117.00 | 2.29 | 0.04 | 0.70 |
| County + ElevDiff : Season + Season : Sex + Season : Timing + Dcoast : Sex | 16 | -542.01 | 1117.00 | 2.33 | 0.04 | 0.73 |
| ElevDiff + County + Season : Sex + Season : Timing | 13 | -545.19 | 1117.10 | 2.34 | 0.04 | 0.77 |
| County + ElevDiff : Season + Season : Sex + Season : Timing + Dcoast : Season + Dcoast : Sex | 17 | -541.07 | 1117.30 | 2.58 | 0.03 | 0.80 |
| County + ElevDiff : Season + ElevDiff : Sex + Season : Sex + Season : Timing | 15 | -543.21 | 1117.30 | 2.61 | 0.03 | 0.83 |
| ElevDiff + County + Dcoast + Season : Sex | 12 | -546.47 | 1117.50 | 2.80 | 0.03 | 0.86 |
| County + Sex + ElevDiff : Season + Season : Timing | 13 | -545.42 | 1117.50 | 2.81 | 0.03 | 0.89 |
| County + Dcoast + ElevDiff : Season + Season : Sex + Season : Timing + Timing : Sex | 16 | -542.32 | 1117.70 | 2.95 | 0.03 | 0.92 |
| ElevDiff + County + Season : Sex + Season : Timing + Dcoast : Season | 15 | -543.40 | 1117.70 | 2.98 | 0.03 | 0.95 |
| County + Timing + Dcoast + ElevDiff : Season + Season : Sex | 14 | -544.46 | 1117.70 | 2.98 | 0.03 | 0.97 |
| County + Dcoast + ElevDiff : Season + ElevDiff : Sex + Season : Sex | 14 | -544.46 | 1117.70 | 2.99 | 0.03 | 1.00 |

| **(c) Models explaining duration of migration** | ***df*** | ***logLik*** | ***AICc*** | ***∆AICc*** | **AICcW** | **CumW** |
| --- | --- | --- | --- | --- | --- | --- |
| Sex + ElevDiff : Season + DS&W : Season + DS&W : Timing + Season : Timing + Dcoast : Season | 15 | -603.07 | 1237.00 | 0.00 | 0.09 | 0.09 |
| ElevDiff : Season + DS&W : Season + DS&W : Timing + Season : Timing + Dcoast : Season + Dcoast : Sex | 16 | -602.07 | 1237.20 | 0.12 | 0.09 | 0.18 |
| ElevDiff : Season + DS&W : Season + DS&W : Timing + Season : Sex + Season : Timing + Dcoast : Season | 16 | -602.19 | 1237.40 | 0.36 | 0.08 | 0.26 |
| ElevDiff : Season + DS&W : Season + DS&W : Timing + Season : Sex + Season : Timing + Dcoast : Season + Dcoast : Sex | 17 | -601.14 | 1237.40 | 0.40 | 0.08 | 0.34 |
| ElevDiff : Season + DS&W : Season + DS&W : Timing + Season : Timing + Dcoast : Season + Sex : Timing | 16 | -602.63 | 1238.30 | 1.25 | 0.05 | 0.39 |
| ElevDiff : Season + ElevDiff : Sex + DS&W : Season + DS&W : Timing + Season : Timing + Dcoast : Season | 16 | -602.71 | 1238.40 | 1.41 | 0.05 | 0.43 |
| ElevDiff : Season + DS&W : Season + DS&W : Timing + Season : Sex + Season : Timing + Dcoast : Season + Sex : Timing | 17 | -601.77 | 1238.70 | 1.66 | 0.04 | 0.48 |
| ElevDiff : Season + DS&W : Season + DS&W : Timing + Season : Timing + Dcoast : Season + Sex : TimingDcoast : Sex | 17 | -601.79 | 1238.70 | 1.69 | 0.04 | 0.52 |
| **Sex + Dcoast + ElevDiff : Season + DS&W : Season + DS&W : Timing + Season : Timing** | **14** | **-605.04** | **1238.90** | **1.84** | **0.04** | **0.55** |
| ElevDiff : Season + ElevDiff : Sex + DS&W : Season + DS&W : Timing + Season : Timing + Dcoast : Season + Dcoast : Sex | 17 | -601.87 | 1238.90 | 1.86 | 0.04 | 0.59 |
| ElevDiff : Season + ElevDiff : Sex + DS&W : Season + DS&W : Timing + Season : Sex + Season : Timing + Dcoast : Season | 17 | -601.90 | 1238.90 | 1.91 | 0.04 | 0.63 |
| ElevDiff : Season + DS&W : Season + DS&W : Sex + DS&W : Timing + Season : Timing + Dcoast : Season + Dcoast : Sex | 17 | -601.90 | 1239.00 | 1.92 | 0.04 | 0.66 |
| ElevDiff : Season + DS&W : Season + DS&W : Sex + DS&W : Timing + Season : Timing + Dcoast : Season | 16 | -603.00 | 1239.00 | 1.98 | 0.04 | 0.70 |
| ElevDiff : Season + DS&W : Season + DS&W : Timing + Season : Sex + Season : Timing + Dcoast : Season + Sex : Timing + Dcoast : Sex | 18 | -600.88 | 1239.10 | 2.02 | 0.03 | 0.73 |
| Sex + ElevDiff : Season + DS&W : Season + DS&W : Timing + Season : Timing | 13 | -606.23 | 1239.10 | 2.11 | 0.03 | 0.76 |
| ElevDiff : Season + DS&W : Season + DS&W : Timing + Season : Timing + Dcoast : Sex | 15 | -604.17 | 1239.20 | 2.20 | 0.03 | 0.79 |
| ElevDiff : Season + DS&W : Season + DS&W : Sex + DS&W : Timing + Season : Sex + Season : Timing + Dcoast : Season + Dcoast : Sex | 18 | -600.98 | 1239.20 | 2.20 | 0.03 | 0.83 |
| ElevDiff : Season + ElevDiff : Sex + DS&W : Season + DS&W : Timing + Season : Sex + Season : Timing + Dcoast : Season + Dcoast : Sex | 18 | -601.00 | 1239.30 | 2.26 | 0.03 | 0.86 |
| ElevDiff + Sex +DS&W : Season + DS&W : Timing + Season : Timing + Dcoast : Season | 14 | -605.26 | 1239.30 | 2.26 | 0.03 | 0.89 |
| ElevDiff : Season + DS&W : Season + DS&W : Sex + DS&W : Timing + Season : Sex + Season : Timing + Dcoast : Season | 17 | -602.12 | 1239.40 | 2.35 | 0.03 | 0.91 |
| Dcoast + ElevDiff : Season + DS&W : Season + DS&W : Timing + Season : Sex + Season : Timing | 15 | -604.25 | 1239.40 | 2.37 | 0.03 | 0.94 |
| ElevDiff + DS&W : Season + DS&W : Timing + Season : Sex + Season : Timing + Dcoast : Season | 15 | -604.26 | 1239.40 | 2.38 | 0.03 | 0.97 |
| ElevDiff + Sex +DS&W : Season + DS&W : Timing + Season : Timing + Dcoast : Season + Dcoast : Sex | 15 | -604.31 | 1239.50 | 2.48 | 0.03 | 1.00 |

| **(d) Models explaining mean travel speed during migration** | ***df*** | ***logLik*** | ***AICc*** | ***∆AICc*** | **AICcW** | **CumW** |
| --- | --- | --- | --- | --- | --- | --- |
| ElevDiff : Season + DS&W : Season + DS&W : Timing + Season : Sex + Dcoast : Season + Sex : Timing | 16 | -336.504 | 706 | 0.00 | 0.101 | 0.101 |
| **ElevDiff : Season + DS&W : Season + DS&W : Timing + Season : Sex + Sex : Timing** | **14** | **-338.831** | **706.4** | **0.42** | **0.082** | **0.183** |
| ElevDiff : Season + DS&W : Season + DS&W : Timing + Season : Sex + Dcoast : Season + Sex : Timing + Sex : Dcoast | 17 | -335.716 | 706.6 | 0.55 | 0.077 | 0.26 |
| ElevDiff : Season + DS&W : Season + DS&W : Sex + DS&W : Timing + Season : Sex + Dcoast : Season + Sex : Timing | 17 | -335.716 | 706.6 | 0.55 | 0.077 | 0.337 |
| ElevDiff : Season + DS&W : Season + DS&W : Sex + DS&W : Timing + Season : Sex + Dcoast : Season + Sex : Timing + Sex : Dcoast | 18 | -334.683 | 706.7 | 0.63 | 0.074 | 0.411 |
| ElevDiff : Season + DS&W : Season + DS&W : Sex + DS&W : Timing + Season : Sex + Sex : Timing | 15 | -338.05 | 707.00 | 0.97 | 0.062 | 0.473 |
| ElevDiff : Season + DS&W : Season + DS&W : Timing + Season : Sex + Season : Timing + Sex : Timing | 15 | -338.296 | 707.5 | 1.46 | 0.049 | 0.522 |
| Dcoast + ElevDiff : Season + DS&W : Season + DS&W : Timing + Season : Sex + Season : Timing + Dcoast : Season + Sex : Timing | 17 | -336.214 | 707.6 | 1.55 | 0.047 | 0.569 |
| ElevDiff : Season + ElevDiff : Sex + DS&W : Season + DS&W : Timing + Season : Sex + Dcoast : Season + Sex : Timing | 17 | -336.42 | 708.00 | 1.96 | 0.038 | 0.607 |
| ElevDiff : Season + DS&W : Season + DS&W : Timing + Season : Sex + Sex : Timing | 15 | -338.557 | 708.00 | 1.98 | 0.038 | 0.645 |
| ElevDiff : Season + DS&W : Season + DS&W : Sex + DS&W : Timing + Season : Sex + Season : Timing + Sex : Timing | 16 | -337.51 | 708.00 | 2.01 | 0.037 | 0.682 |
| Dcoast + ElevDiff : Season + DS&W : Season + DS&W : Sex + DS&W : Timing + Season : Sex + Season : Timing + Dcoast : Season + Sex : Timing | 18 | -335.42 | 708.10 | 2.10 | 0.04 | 0.72 |
| Dcoast + ElevDiff : Season + DS&W : Season + DS&W : Timing + Season : Sex + Season : Timing + Dcoast : Season + Sex : Timing + Sex : Dcoast | 18 | -335.48 | 708.20 | 2.21 | 0.03 | 0.75 |
| Dcoast + ElevDiff:Season + DS&W:Season + DS&W:Sex + DS&W:Timing + Season:Sex + Season:Timing + Dcoast:Season + Sex:Timing + Sex:Dcoast | 19 | -334.44 | 708.30 | 2.29 | 0.03 | 0.78 |
| ElevDiff : Season + ElevDiff : Sex + DS&W : Season + DS&W : Timing + Season : Sex + Sex : Timing | 15 | -338.75 | 708.40 | 2.38 | 0.03 | 0.82 |
| Dcoast + ElevDiff : Season + DS&W : Season + DS&W : Sex + DS&W : Timing + Season : Sex + Sex : Timing | 16 | -337.74 | 708.50 | 2.48 | 0.03 | 0.84 |
| ElevDiff : Season + ElevDiff : Sex + DS&W : Season + DS&W : Sex + DS&W : Timing + Season : Sex + Dcoast : Season + Sex : Timing | 18 | -335.68 | 708.70 | 2.62 | 0.03 | 0.87 |
| ElevDiff : Season + ElevDiff : Sex + DS&W : Season + DS&W : Timing + Season : Sex + Dcoast : Season + Sex : Timing + Sex : Dcoast | 18 | -335.69 | 708.70 | 2.64 | 0.03 | 0.90 |
| ElevDiff : Season + DS&W : Season + DS&W : Timing + Season : Sex + Sex : Timing + Sex : Dcoast | 16 | -337.85 | 708.70 | 2.69 | 0.03 | 0.92 |
| ElevDiff : Season + DS&W : Season + DS&W : Sex + DS&W : Timing + Season : Sex + Sex : Timing + Sex : Dcoast | 17 | -336.80 | 708.80 | 2.72 | 0.03 | 0.95 |
| ElevDiff : Season + ElevDiff : Sex + DS&W : Season + DS&W : Sex + DS&W : Timing + Season : Sex + Dcoast : Season + Sex : Timing + Sex : Dcoast | 19 | -334.68 | 708.80 | 2.77 | 0.03 | 0.98 |
| Dcoast + ElevDiff : Season + DS&W : Season + DS&W : Timing + Season : Sex + Season : Timing + Sex : Timing | 16 | -337.99 | 709.00 | 2.97 | 0.02 | 1.00 |

| **(e) Models explaining the use of stopover** | ***df*** | ***logLik*** | ***AICc*** | ***∆AICc*** | **AICcW** | **CumW** |
| --- | --- | --- | --- | --- | --- | --- |
| ElevDiff + DS&W : Season + DS&W : Timing + Season : Sex + Sex : Timing | 12 | -254.52 | 533.60 | 0.00 | 0.15 | 0.15 |
| **ElevDiff + DS&W : Season + DS&W : Timing + Sex : Timing** | **11** | **-255.69** | **533.90** | **0.25** | **0.13** | **0.29** |
| ElevDiff : Sex + DS&W : Season + DS&W : Timing + Sex : Timing | 12 | -255.15 | 534.90 | 1.26 | 0.08 | 0.37 |
| ElevDiff + DS&W : Season + DS&W : Timing + DS&W : Sex + Season : Sex + Sex : Timing | 13 | -254.13 | 534.90 | 1.33 | 0.08 | 0.45 |
| ElevDiff + DS&W : Season + DS&W : Timing + DS&W : Sex + Sex : Timing | 12 | -255.20 | 535.00 | 1.37 | 0.08 | 0.52 |
| ElevDiff : Sex + DS&W : Season + DS&W : Timing + Season : Sex + Sex : Timing | 13 | -254.20 | 535.10 | 1.45 | 0.07 | 0.60 |
| ElevDiff + Dcoast + DS&W : Season + DS&W : Timing + Season : Sex + Sex : Timing | 13 | -254.21 | 535.10 | 1.48 | 0.07 | 0.67 |
| ElevDiff + Dcoast + DS&W : Season + DS&W : Timing + Sex : Timing | 12 | -255.33 | 535.20 | 1.62 | 0.07 | 0.74 |
| ElevDiff : Season + DS&W : Season + DS&W : Timing + Season : Sex + Sex : Timing | 13 | -254.36 | 535.40 | 1.78 | 0.06 | 0.80 |
| ElevDiff : Season + DS&W : Season + DS&W : Timing + Sex : Timing | 12 | -255.54 | 535.70 | 2.04 | 0.06 | 0.86 |
| ElevDiff + DS&W : Season + DS&W : Timing + Season : Timing + Season : Sex + Sex : Timing | 13 | -254.51 | 535.70 | 2.08 | 0.05 | 0.91 |
| ElevDiff + DS&W : Season + DS&W : Timing + Season : Timing + Sex : Timing | 12 | -255.69 | 536.00 | 2.34 | 0.05 | 0.96 |
| ElevDiff : Sex + DS&W : Season + DS&W : Timing + DS&W : Sex + Sex : Timing | 13 | -254.71 | 536.10 | 2.48 | 0.04 | 1.00 |

**Table S3.2.** Model selection tables for proportion of daily time active. The full model of the first model set included fixed effects of use of stopover (3 categories: no use of stopover, use of stopover - day at stopover, use of stopover - day in travel) season (spring *vs.* autumn), sex, county, distance of the winter home range barycenter to coastline (‘Dcoast’), total distance roamed during migration, duration and timing of departure (‘Timing’), and their two-way interactions with sex and season. In addition, a fixed effect for collar brand was added to account for potential differences. Individual identities and years were added as random factors in all models. The loglikelihood (LogLik), corresponding AIC*c* weight (AIC*cW*) are presented for all models with a ΔAIC*c* < 2.7. The candidate model sets (i.e., models with a ΔAIC*c* < 2) are in grey shade and the selected models are shown in bold.

| **Models explaining proportion of time active** | ***df*** | ***logLik*** | ***AICc*** | ***∆AICc*** | **AICcW** | **CumW** |
| --- | --- | --- | --- | --- | --- | --- |
| Duration + Stopover + County : Season + Season : Sex + Timing : Season + Timing : Sex | 19 | 2652.19 | -5266.10 | 0.00 | 0.23 | 0.23 |
| Collar + Duration + Stopover + County : Season + Season : Sex + Timing : Season + Timing : Sex | 20 | 2652.62 | -5264.90 | 1.17 | 0.13 | 0.35 |
| **Duration + Stopover + County : Season + Season : Sex + Timing : Season** | **18** | **2650.39** | **-5264.50** | **1.58** | **0.10** | **0.46** |
| Duration + County : Season + Season : Sex + Timing : Season + Stopover : Sex +Timing : Sex | 21 | 2653.38 | -5264.40 | 1.69 | 0.10 | 0.56 |
| Stopover + County : Season + Duration : Sex + Season : Sex + Timing : Season + Timing : Sex | 20 | 2652.24 | -5264.10 | 1.93 | 0.09 | 0.64 |
| Duration + Stopover + Dcoast +County : Season + Season : Sex + Timing : Season + Timing : Sex | 20 | 2652.22 | -5264.10 | 1.97 | 0.09 | 0.73 |
| Duration + County : Season + Season : Sex + Timing : Season + Stopover : Sex | 20 | 2651.88 | -5263.40 | 2.66 | 0.06 | 0.79 |

**Appendix - S4. Predictor weights**

**Table S4.1. Model predictor weights.** Predictor weights calculated as the sum of the Akaike weights for each model in which that variable appeared for the five analyses performed on migration characteristics and the one on daily characteristic. Variables retained in the selected model are in bold. Deer identity and year were included as random factors in all models.

| **Covariate (fixed effect)** | **Departure timing** | **Distance roamed** | **Duration** | **Speed** | **Stop-over use** | **Daily time active** |
| --- | --- | --- | --- | --- | --- | --- |
| Collar brand | - | - | - | - | - | 0.37 |
| Departure timing | - | 0.66 | **1.00** | **1.00** | **1.00** | **0.98** |
| Departure timing : sex | - | 0.22 | 0.35 | **0.99** | **0.95** | 0.62 |
| Departure timing : season | - | 0.47 | **0.96** | 0.34 | 0.26 | **0.97** |
| Distance roamed | - | - | - | - | - | <0.01 |
| Distance roamed : sex | - | - | - | - | - | <0.01 |
| Duration | - | - | - | - | - | **0.99** |
| Duration : sex | - | - | - | - | - | 0.30 |
| Stopover use | - | - | - | - | - | **1.00** |
| Stopover use : sex | - | - | - | - | - | 0.32 |
| Stopover use : season | - | - | - | - | - | 0.13 |
| Distance between seasonal ranges | 0.56 | - | **1.00** | **1.00** | **1.00** | - |
| Distance between seasonal ranges : sex | 0.10 | - | 0.28 | 0.46 | 0.37 | - |
| Distance between seasonal ranges : season | 0.15 | - | **0.80** | **1.00** | **0.79** | - |
| Distance between seasonal ranges : departure timing | - | - | **1.00** | **0.97** | **0.77** | - |
| Absolute difference in elevation during migration | 0.58 | **1.00** | **1.00** | **0.99** | **1.00** | - |
| Absolute difference in elevation during migration : sex | 0.16 | 0.26 | 0.30 | 0.26 | 0.35 | - |
| Absolute difference in elevation during migration : season | 0.35 | 0.69 | **0.79** | **0.93** | 0.29 | - |
| Winter range distance to coast | **1.00** | 0.63 | **0.88** | 0.75 | 0.52 | 0.51 |
| Winter range distance to coast : sex | 0.20 | 0.23 | 0.40 | 0.33 | 0.17 | 0.14 |
| Winter range distance to coast : season | **0.98** | 0.26 | 0.63 | 0.53 | 0.16 | 0.26 |
| County | **0.99** | **0.96** | 0.10 | 0.12 | 0.18 | **0.80** |
| County : season | **0.91** | 0.15 | 0.01 | 0.06 | 0.03 | **0.79** |
| Season : sex | 0.16 | **0.78** | 0.47 | **1.00** | 0.50 | **0.97** |
| Season | **1.00** | **1.00** | **1.00** | **1.00** | **1.00** | **1.00** |
| Sex | 0.57 | **1.00** | **1.00** | **1.00** | **1.00** | **1.00** |

**Appendix - S5. Result section, details**

***Individual migration characteristics***

*(i) Timing of departure*

Departure timing varied between counties and with the distance of the winter home range barycentre to coastline, but these effects depended on season (Table 3). Individuals having their winter home range further away from the coastline left their seasonal range later (i.e. home ranges 28 km further from the coast left 1.4 and 10 days later in the spring and autumn, respectively), with a stronger effect in autumn (Table 3; Fig S5.1).

**B**


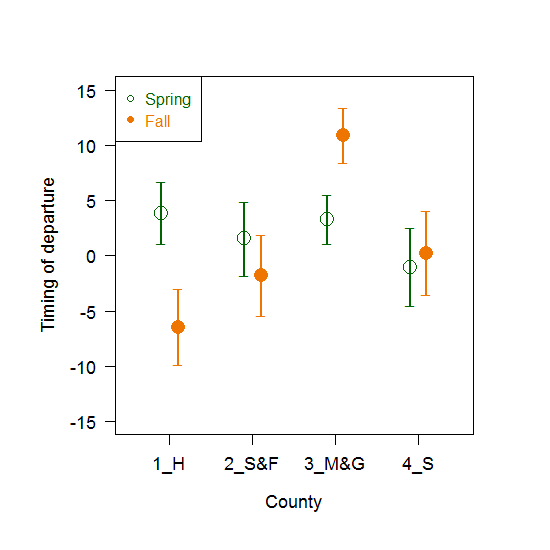


Autumn

**A**

Spring

Hordaland

Sogn & Fjordane

Møre & Romsdal

Sør-Trøndelag

**Figure S5.1.** Relationships between migration timing of departure and (A) winter range distance to coastline or (B) county of capture, for both seasons according to the selected model. The lines or points depict relationships predicted by the selected models along with their corresponding standard error.

*(ii) Distance roamed during migration*

The total distance roamed during migration increased as the absolute difference in elevation increased, e.g. a 550 m increase in elevation increased the total distance roamed during migration of 7.76 km (Table 3; Fig. 1; Fig S5.2). The total distance roamed during migration was 9.51 km longer for females and 6.55 km longer for males in spring compared to autumn, and 9.27 km longer for males compared to females in spring and 4.45 km in autumn (Table 3; Fig. 1; Fig S5.2).

**Figure S5.2.** Relationship between the total distance roamed during migration and the absolute difference in elevation during migration. The line depicts the relationship predicted by the selected model along with their corresponding standard error.

*(iii) Duration of migration*

The duration of migration increased as the individual distance of the winter home range barycenter to coastline increased, e.g. individuals with winter ranges 140 km further from coast spent 1.9 day longer migrating. The duration of migration lasted 3.5 days longer in spring compared to autumn and 4 days longer for males compared to females (Table 3; Fig 1; Fig S5.3). The duration also increased as the difference in elevation increased, but only in spring, where a 550 m increase in elevation difference led to 3 and 0.5 days longer spring and autumn migrations, respectively (Table 3; Fig S5.3). In spring, the duration decreased by 2 days as the timing of departure became 10 days later, while it decreased only by 0.8 day in autumn migration. Finally, duration increased as the distance between seasonal ranges increased, especially for individual leaving early and in spring, with a linear distance between seasonal ranges increasing of 10 km leading to a 5.2 days longer migration (Table 3; Fig S5.3).

Autumn

Autumn

Autumn

Spring

Spring

**A**

**D**

**C**

**B**

Spring

**Figure S5.3.** Relationships between migration duration and (A) winter range distance to coastline, (B) absolute difference in elevation during migration, (C) linear distance between seasonal ranges or (D) timing of departure. The lines depict the relationship predicted by the selected models along with their corresponding standard error.

*(iv) Travel speed*

Autumn migration was 0.02 and 0.15 km/h faster than in spring in females and males, respectively; and females migrated faster than males, especially during spring (0.25 and 0.12 km/h faster in spring and autumn respectively; Table 3; Fig 1). Mean migration speed increased as individuals left later, especially for males, where a 10 days later departure led to 0.02 km/h faster speed. Increasing distance between seasonal ranges also led to faster migration speed, especially in the autumn and for individuals with later departure, while travel speed decreased with increasing elevation difference, where a 550 m increase in elevation decreased females’ speed to 0.14 km/h in the spring and 0.01 km/h in the autumn (Table 3; Fig S5.4).

**B**

**A**

Autumn

Autumn

**C**

Spring

Spring

**Figure S5.4.** Relationships between mean travel speed during migration and (A) absolute difference in elevation during migration, (B) linear distance between seasonal ranges or (C) timing of departure. The lines depict the relationship predicted by the selected models along with their corresponding standard error.

Autumn

Spring

*(v) Use of stopover sites*

The probability of using stopover sites was higher during spring and for males, increased with increasing difference in elevation and with increasing distance between seasonal ranges, especially during spring (Table 3; Fig. 1; Fig S5.5). A 550 m increase in elevation difference increased the probability of using stopovers by 11%; while in spring, a linear distance between seasonal ranges increasing of 10 km, increased the probability by 6.5%. The use of stopover sites decreased with later departure in males only (Table 3), where a 86% decline of the use of stopover site was found throughout the migration period, while the females use of stopover remained stable (Table 3; Fig S5.5).

**B**

**A**

**
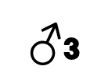

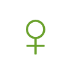
**  **Figure** **S5.5.** Relationships between the probability of using the stopover sites during migration (no use = 0, low value; use = 1, high value) and (A) absolute difference in elevation during migration, (B) linear distance between seasonal ranges or (C) timing of departure. The lines depict the relationship predicted by the selected models along with their corresponding standard error

Spring

Autumn

**C**

*(vi) Time spent active*

Time active was 3.3% higher for females than males and was higher during spring compared to autumn but the magnitude of the effect depended on the county (Table 4; Fig. 2). Time active decreased with decreasing duration of migration and later departure in autumn, but increased with later departure in spring (Table 4; Fig S5.6), e.g. a 10 day later departure led to a 0.4% increase and 0.3% decrease in activity ion spring and autumn, respectively. Individuals using stopovers showed 2.23% higher activity when travelling compared to individuals not using stopover sites (Table 4; Fig 2).

**B**

**A**

Autumn

Spring

Duration of migration (days)

**Figure S5.6.** Relationships between the proportion of daily time spent active during migration and (A) the duration of migration or (B) timing of departure. The lines depict the relationship predicted by the selected models along with their corresponding standard error.

**Appendix – S6. Use of a Multivariate ANalyses Of Variance (MANOVA)**

Since some of the migration traits we have considered are correlated (as shown in appendix S2 for details); and especially the total distance roamed and the duration of migration, the use of MANOVA would have been a possible approach to investigate which factors (sex, environment) were linked to those migration traits. Unfortunately, a MANOVA cannot include random factors, and in our case we have repeated observations for individuals and year. We have performed a MANOVA (without random terms) in order to compare the results from this analysis with the GLMMs we had performed (Table S6.1). The results of the MANOVA are very similar to the GLMMs, and the conclusions are virtually the same (Table S6.1), allowing us to be confident with the results from the GLMMs approach. However, a major disadvantage to this method is that the exact same dependent variables are tested for each independent variable, so that the effect of timing of departure cannot be tested on the other migration traits.

|  | ***Departure timing*** | | | | |  | ***Total distance roamed*** | | | | |  | ***Duration*** | | | | |
| --- | --- | --- | --- | --- | --- | --- | --- | --- | --- | --- | --- | --- | --- | --- | --- | --- | --- |
| **Model variable (fixed effect)** | **Df** | **Sum Sq** | **Mean Sq** | **F value** | **Pr(>F)** |  | **Df** | **Sum Sq** | **Mean Sq** | **F value** | **Pr(>F)** |  | **Df** | **Sum Sq** | **Mean Sq** | **F value** | **Pr(>F)** |
| Distance between seasonal ranges | 1 | 1806 | 1806.5 | 3.12 | 0.078 |  | **1** | **264.81** | **264.81** | **1064.75** | **< 2.2e-16** |  | **1** | **114.33** | **114.33** | **183.0** | **< 2.2e-16** |
| season | 1 | 11 | 11.4 | 0.02 | 0.888 |  | **1** | **4.075** | **4.07** | **16.38** | **0.000** |  | **1** | **15.41** | **15.41** | **24.67** | **0.000** |
| Elevation difference during migration | 1 | 19 | 19.1 | 0.03 | 0.856 |  | **1** | **1.1** | **1.1** | **4.42** | **0.036** |  | **1** | **6.5** | **6.50** | **10.41** | **0.001** |
| sex | 1 | 113 | 113 | 0.20 | 0.659 |  | **1** | **8.676** | **8.67** | **34.88** | **0.000** |  | **1** | **18.69** | **18.69** | **29.92** | **0.000** |
| Distance to coast | **1** | **7124** | **7123.5** | **12.30** | **0.000** |  | 1 | 0.2 | 0.2 | 0.81 | 0.370 |  | 1 | 0.52 | 0.52 | 0.83 | 0.362 |
| county | **3** | **5355** | **1784.9** | **3.08** | **0.027** |  | **3** | **2.479** | **0.82** | **3.32** | **0.020** |  | 3 | 4.66 | 1.55 | 2.48 | 0.060 |
| Distance between ranges : season | 1 | 424 | 424.3 | 0.73 | 0.392 |  | 1 | 0 | 0 | 0.00 | 0.970 |  | **1** | **2.44** | **2.44** | **3.91** | **0.049** |
| Elevation difference : season | 1 | 1239 | 1239.3 | 2.14 | 0.144 |  | 1 | 0.666 | 0.66 | 2.68 | 0.102 |  | **1** | **2.41** | **2.41** | **3.86** | **0.050** |
| Elevation difference : sex | 1 | 1072 | 1072.5 | 1.85 | 0.174 |  | 1 | 0.014 | 0.01 | 0.06 | 0.814 |  | 1 | 0.49 | 0.49 | 0.79 | 0.376 |
| period: Distance to coast | **1** | **4988** | **4987.9** | **8.62** | **0.003** |  | 1 | 0.09 | 0.09 | 0.36 | 0.548 |  | **1** | **2.49** | **2.49** | **3.99** | **0.046** |
| Distance to coast : sex | 1 | 300 | 300 | 0.52 | 0.472 |  | 1 | 0.628 | 0.62 | 2.53 | 0.113 |  | 1 | 1.92 | 1.92 | 3.07 | 0.080 |
| season:county | **3** | **6661** | **2220.4** | **3.84** | **0.010** |  | 3 | 0.388 | 0.12 | 0.52 | 0.669 |  | 3 | 0.38 | 0.12 | 0.20 | 0.896 |
| season:sex | 1 | 251 | 250.9 | 0.43 | 0.511 |  | **1** | **1.03** | **1.03** | **4.14** | **0.042** |  | 1 | 1.1 | 1.10 | 1.76 | 0.185 |
| Residuals | 532 | 307985 | 578.9 |  |  |  | 532 | 132.31 | 0.249 |  |  |  | 532 | 332.37 | 0.625 |  |  |

|  | ***Speed*** | | | | |  | ***Use of stopover*** | | | | |
| --- | --- | --- | --- | --- | --- | --- | --- | --- | --- | --- | --- |
| **Model variable (fixed effect)** | **Df** | **Sum Sq** | **Mean Sq** | **F value** | **Pr(>F)** |  | **Df** | **Sum Sq** | **Mean Sq** | **F value** | **Pr(>F)** |
| Distance between seasonal ranges | **1** | **19.421** | **19.421** | **78.10** | **< 2.2e-16** |  | **1** | **32.696** | **32.696** | **78.88** | **< 2.2e-16** |
| season | **1** | **17.729** | **17.729** | **71.30** | **0.000** |  | **1** | **12.463** | **12.463** | **30.07** | **0.000** |
| Elevation difference during migration | **1** | **2.143** | **2.143** | **8.62** | **0.003** |  | **1** | **4.888** | **4.888** | **11.79** | **0.001** |
| sex | **1** | **45.143** | **45.143** | **181.55** | **< 2.2e-16** |  | **1** | **5.893** | **5.893** | **14.22** | **0.000** |
| Distance to coast | 1 | 0 | 0 | 0.00 | 0.997 |  | 1 | 0.493 | 0.493 | 1.19 | 0.276 |
| county | 3 | 0.095 | 0.032 | 0.13 | 0.944 |  | 3 | 0.63 | 0.21 | 0.51 | 0.678 |
| Distance between ranges : season | **1** | **4.048** | **4.048** | **16.28** | **0.000** |  | **1** | **4.18** | **4.18** | **10.08** | **0.002** |
| Elevation difference : season | **1** | **1.954** | **1.954** | **7.86** | **0.005** |  | **1** | **2.913** | **2.913** | **7.03** | **0.008** |
| Elevation difference : sex | 1 | 0.798 | 0.798 | 3.21 | 0.074 |  | 1 | 0 | 0 | 0.00 | 0.998 |
| period: Distance to coast | **1** | **1.511** | **1.511** | **6.08** | **0.014** |  | 1 | 1.05 | 1.05 | 2.53 | 0.112 |
| Distance to coast : sex | **1** | **0.994** | **0.994** | **4.00** | **0.046** |  | **1** | **1.773** | **1.773** | **4.28** | **0.039** |
| season:county | 3 | 0.139 | 0.046 | 0.19 | 0.905 |  | 3 | 0.837 | 0.279 | 0.67 | 0.569 |
| season:sex | **1** | **8.947** | **8.947** | **35.98** | **0.000** |  | 1 | 0.808 | 0.808 | 1.95 | 0.163 |
| Residuals | 532 | 132.29 | 0.249 |  |  |  | 532 | 220.51 | 0.414 |  |  |

***Table S6.1.*** *Results from the MANOVA analysis including the 5 migration traits as dependent variables and the same independent variables as the GLMMs. Significant variables are presented in bold. A variable significant in the MANOVA but not selected in the previously performed GLMMs is presented in red, and a factor not significant in the MANOVA but selected in the previously performed GLMM is presented in orange. Consistent results for both the MANOVA and GLMMs are in green.*
